# A survey of indoor and outdoor *Anopheles* density, species composition and circumsporozoite rate of malaria vectors on the Bijagós Archipelago, Guinea-Bissau

**DOI:** 10.1186/s12936-025-05372-z

**Published:** 2025-06-19

**Authors:** Elizabeth Pretorius, Robert T. Jones, Harry Hutchins, Eunice Teixeira Da Silva, Sainey Ceesay, Mamadou Ousmane Ndiath, Umberto d’Alessandro, Amabelia Rodrigues, James G. Logan, Anna Last

**Affiliations:** 1 Department of Disease Control, London School of Hygiene & Tropical Medicine, London, UK; 2 Arctech Innovation, Dagenham, London, UK; 3 Clinical Research Department, London School of Hygiene & Tropical Medicine, London, UK; 4 Projecto de Saúde Bandim, Bissau, Guinea-Bissau; 5 Ministério de Saúde Pública, Bissau, Guinea-Bissau; 6 Medical Research Council Unit The Gambia at London School of Hygiene & Tropical Medicine, Fajara, The Gambia

**Keywords:** Malaria, Bijagós Archipelago, Guinea-Bissau, Vector survey, *Anopheles gambiae*

## Abstract

**Background:**

The malaria-endemic Bijagós Archipelago is situated 50 km off the coast of mainland Guinea-Bissau. It is a seasonal malaria transmission setting, with insecticide-treated bed nets as its primary control strategy. Little is known about the vector diversity and behaviour across the Archipelago.

**Methods:**

In 2019, a survey took place on 16 of the inhabited islands across the Archipelago. Adult mosquitoes were collected using odour-baited outdoor light traps and indoor light traps at houses selected at random. Larval surveys were conducted for each village sampled. *Anopheles* adults caught were morphologically identified and a sub-sample was analysed to identify species within the *Anopheles gambiae* complex using RFLP-PCR. Sporozoite positivity was detected within a sub-sample by CSP-ELISA.

**Results:**

*Anopheles gambiae *sensu lato was present on all islands sampled. *Anopheles* density varied between islands, with densities ranging from 0.0 to 98.7 per trapping night from indoor traps and 0.1–165.2 per trapping night from outdoor traps. *Anopheles melas* was the most commonly observed species, accounting for 85.2% of all *Anopheles* caught from both indoor and outdoor light traps. A high level of hybridization between *An. gambiae *sensu stricto and *Anopheles coluzzii* was seen on some islands across the Archipelago. The overall sporozoite rate was 0.86% (0.2% for indoor traps; 1.4% for outdoor traps).

**Conclusions:**

Species within *An. gambiae s.l*. are the primary vectors on the Bijagós. *Anopheles melas* may contribute to transmission throughout the year in the Bijagós. The vector species composition, abundance and infection rates uncovered in this study are useful for informing tailored, effective vector control programme in the Bijagos.

## Background

While great strides have been made in malaria control over the last several decades through the mass distribution of insecticide-based interventions, progress has stalled since 2015 [1, 2]. This is multifactorial, including the observed increase in resistance in both the parasite to artemisinin-combination therapy and vector to insecticide, and residual transmission. Residual transmission refers to transmission that persists regardless of full universal coverage of insecticide-treated nets (ITNs) and/or indoor residual spraying (IRS), which contain active ingredients that are effective against fully susceptible local vector populations [3].

Current mainstay vector control measures target endophilic (resting indoors) and night biting mosquitoes. This enables mosquitoes that display different behaviours to evade contact with insecticide-treated surfaces. These behaviours include resting and/or feeding outdoors, feeding earlier in the evening or later in the morning when human hosts are not protected by bed nets, or not preferentially feeding on humans [3]. Between- and within- species variation in feeding behaviours has been well documented [4]. The diversity of vectors and their behaviours in each setting will impact the ability of interventions to effectively control malaria. It is, therefore, important to thoroughly understand the vector population prior to control measures being implemented.

The Bijagós Archipelago consists of 88 islands and islets which lie approximately 50 km off the coast of mainland Guinea-Bissau. Nineteen of the islands are permanently inhabited and home to approximately 25,000 people [5]. The population mainly consists of subsistence farmers and fishermen [6]. The islands’ populations are relatively isolated, with travel between island usually being made to seek health care, attend cultural festivities or family events, or for income-generating and subsistence activities [6]. This isolation and the geographic topography of the islands makes it an ideal location for investigating vector control strategies [7].

Malaria on the Bijagós Archipelago is seasonal, primarily occurring during the rainy season in June to December, with peak prevalence in November [8]. Malaria vector control is almost exclusively reliant on the distribution of ITNs, with triennial bed net distribution, the last of which was in 2017. In 2017, surveys were conducted on the island of Bubaque, the most heavily populated island in the Bijagós [8]. The survey identified *Anopheles gambiae *sensu stricto (*s.s*.) as the primary vector on Bubaque during the transmission season, and *Anopheles melas* was thought to be responsible for the low level of transmission that occurs during the dry season. Here, *Anopheles* density, species composition and circumsporozoite data of malaria vectors from across the Archipelago were recorded to determine whether the previous findings on Bubaque are representative of vector populations throughout Bijagós, and better inform future control programmes on the islands.

## Methods

### Study site

**Fig. 1:**
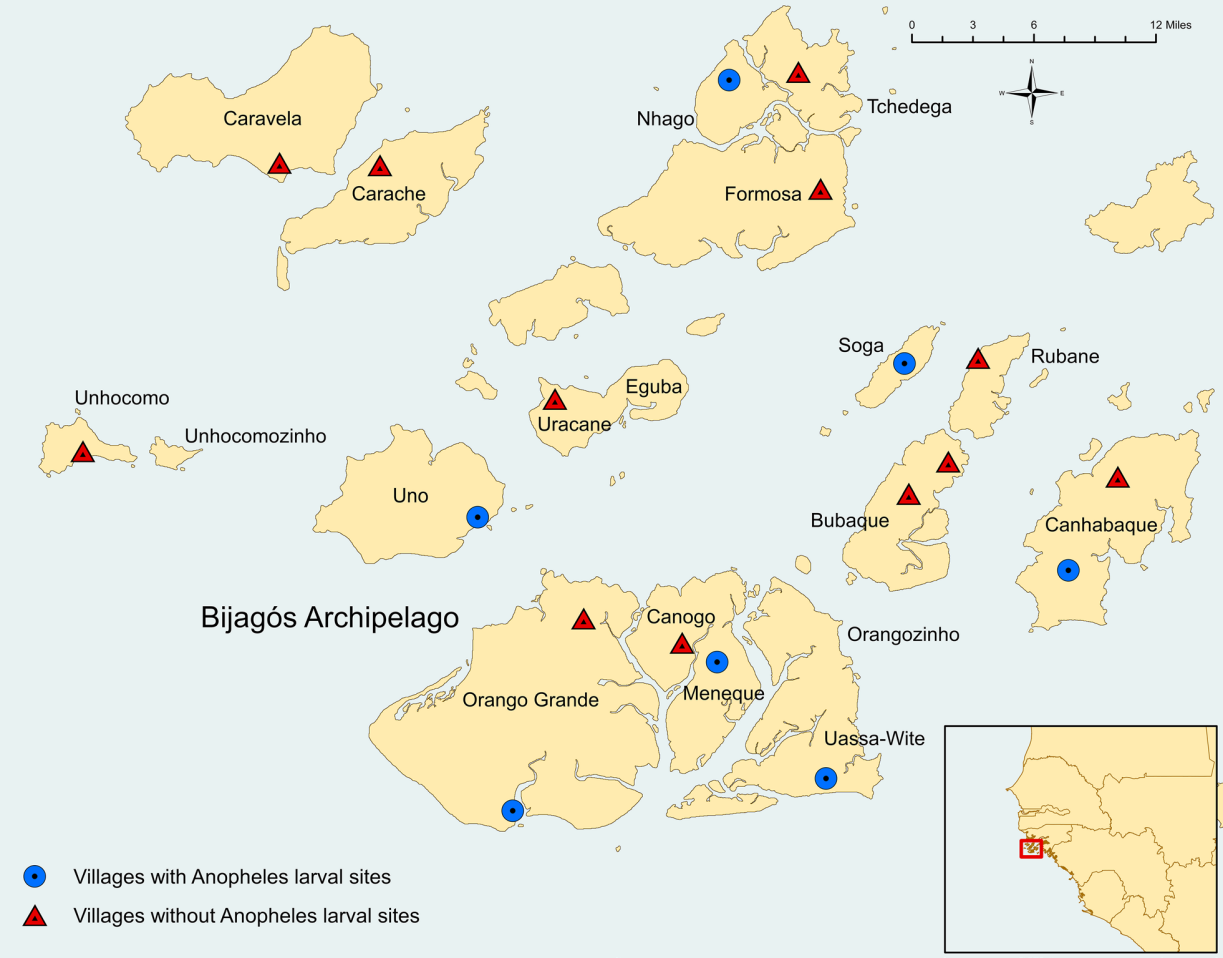
Map of the Bijagós Archipelago showing villages and *Anopheles* larval sites sampled in 2019. Location of the Archipelago in West Africa shown in inset

The survey was a cross-sectional survey that took place on 16 of the inhabited islands on the Bijagós Archipelago during a single peak transmission season (Fig. 1). A two-stage sampling process was employed, where villages were sampled during the baseline prevalence survey for a cluster randomized trial using probability proportional to size sampling methodology [9]. All villages selected for the baseline survey were assigned a unique code, and from this, one to two villages per island were randomly selected for entomology sampling using a random number generator to select from the list of coded villages. Head of household lists for each of the selected villages were used to randomly select households for indoor trapping.

### Adult trapping and molecular analysis

Adult mosquitoes were caught using both indoor and outdoor CDC Light traps (LTs; John W. Hock, Gainesville, Florida, USA) in eight houses on each island sampled. Verbal consent was given prior to LT set-up. Indoor LTs were used to capture mosquitoes that rest or feed indoors, targeting species that are endophilic or endophagic, and outdoor LTs were used to capture exophilic or exophagic mosquitoes. These are safer and more ethical alternatives to human landing catches, which expose participants to potentially-infectious mosquitoes.

One indoor LT per house was placed 50 cm from the base of the bed at a height of 100 cm [10]. Outdoor LTs were placed 5–10 m from the house in a clear area at a height of 100 cm. Outdoor LTs were baited using the MB5-lure blend; these lures were hung from the side bracket of the trap using wire [11]. Carbon dioxide was also produced for the outdoor LTs using a 17.5 g dried active yeast with 250 g sugar in 2.5 L water [12]. Both indoor and outdoor LTs ran from 19 h00 to 07 h00 for two consecutive nights. Following each nights’ trapping, mosquitoes were killed using acetone and morphologically identified [13]. Mosquitoes were then individually dry-preserved in self-indicating silica gel in 1.5 ml microcentrifuge tubes. Mosquito density was calculated as the number of mosquitoes caught divided by the number of trapping nights (number of houses multiplied by the number of nights trapped) for each island.

A subsample of 100 *An. gambiae *sensu lato (*s.l.)* from indoor and outdoor LTs from each island underwent further molecular analysis. To identify species within *An. gambiae s.l*., DNA was extracted using the automated QIA cube Extractor robot (Qiagen, Hilden, Germany) following manufacturer instructions. PCR was then performed using restriction fragment length polymorphism (RFLP) [14]. To test for the presence of the circumsporozoite protein (CSP), the head and thorax of *An. gambiae* were ground in 1.5 ml microcentrifuge tubes using BB NP40 solution. An enzyme-linked immunosorbent assay (ELISA) was performed on mosquito triturate [15].

### Larval survey

A larval survey was conducted for every village sampled by searching the surrounding area for suitable mosquito larval habitats. Larval habitats were characterized, and environmental variables were recorded. Variables included size of larval site perimeter, presence/absence of direct sunlight, the presence/absence of vegetation and vegetation type, and habitat type (grouped into natural or man-made). Natural habitats included rain pools, drainage channels and erosion pits; artificial habitats included drainage pools from wells, cut-out palm tree hollows, salt pits and borrow pits [16]. Larval habitats were grouped into four size categories: 0.01–1 m, 1.01–10 m, 10.01–100 m and > 100 m [8]. The size of the larval habitat dictated the number of dips per site; 3, 5, 15 or 50 dips, respectively. Dipping techniques varied depending on larvae habitat, such as partial submersion of larval dipper around emergent vegetation, logs and tree stumps. Full sampling techniques have been previously described [17]. Larvae were collected and identified to be within the anopheline or culicine subfamily. Larval density and proximity to selected villages was recorded.

## Results

### Mosquito density and species composition

A total of 8625 female mosquitoes were caught by LT; 6229 (72%) were *Anopheles*, 2391 (28%) were *Culex* genus and five (0.06%) were in the *Aedes* genus. Of the 6229 *Anopheles* caught, 2760 were from indoor LTs and 3469 from outdoor LTs. All *Anopheles* females were morphologically identified as being within the *An. gambiae* complex.

**Fig. 2:**
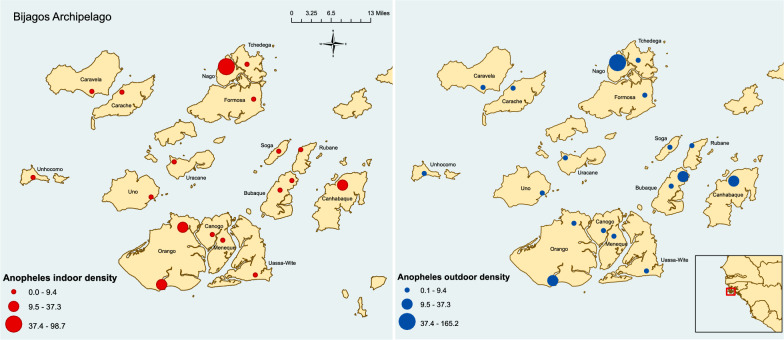
*Anopheles* density (number per trapping night) from indoor (red) and outdoor (blue) LTs across the Bijagós Archipelago

The number of *Anopheles* females caught varied considerably between islands from both indoor and outdoor trapping (Fig. 2). For indoor trapping, the density of *Anopheles* females caught ranged from none on Unhocomo island to 98.7 per trapping night on Nhago island. For outdoor trapping, density ranged from 0.1 per trapping night on a few islands to 165.2 per trapping night on Nhago (Table 1). Except for the islands of Carache, Caravela and Unhocomo, *Anopheles* females dominated trap catches from both indoor and outdoor LTs.

**Table 1:** Total number of trapping nights, total female *Anopheles* caught, density and percentage of *Anopheles* females in trap catch for each island sampled using indoor and outdoor LTs

Island	Indoor trapping			Outdoor trapping		
	Total trapping nights	Total *Anopheles* females (% of trap catch^a^)	*Anopheles* density^b^	Total nights trapped	Total *Anopheles* females (% of trap catch^a^)	*Anopheles* density^b^
Bubaque	16	104 (50.2)	6.5	13	110 (83.3)	8.5
Canhabaque	13	278 (71.1)	21.4	14	391 (76.8)	27.9
Canogo	16	77 (62.6)	4.8	16	44 (50.0)	2.9
Carache	16	5 (2.7)	0.3	16	1 (1.5)	0.1
Caravela	15	11 (4.1)	0.7	16	4 (2.4)	0.2
Formosa	15	19 (19.6)	1.3	16	2 (6.1)	0.1
Meneque	7	25 (46.3)	3.6	8	39 (73.6)	4.9
Nhago	17	1678 (95.2)	98.7	15	2478 (96.7)	165.2
Orango Grande	15	374(88.6)	24.9	17	201 (84.4)	11.8
Rubane	15	46 (65.7)	3.1	15	56 (68.3)	3.7
Soga	15	19 (13.3)	1.3	16	31 (40.8)	1.9
Tchedega	15	69 (51.9)	4.6	15	5 (14.7)	0.3
Uassa-Wite	13	36 (48.0)	2.8	14	81 (71.0)	5.8
Unhocomo	15	0 (0.0)	0.0	16	2 (3.2)	0.1
Uno	16	12 (11.3)	0.7	16	12 (22.6)	0.7
Uracane	16	7 (7.1)	0.4	16	12 (11.8)	0.7

^a^Percentage of mosquitoes caught that were *Anopheles* females

^b^Total *Anopheles* females per trap per night

A total of 902 *An. gambiae* specimens (409 from indoor LTs, 431 from outdoor LTs) were successfully amplified and their species identified. *Anopheles melas* dominated, accounting for 85.2% of all *An. gambiae s.l.* caught, followed by *An. gambiae s.s*./*Anopheles coluzzii* hybrids (7.0%), *An. gambiae s.s.* (6.1%) and *An. coluzzii* (1.7%). This was consistent throughout most islands sampled (Table 2). Formosa, Uassa-Wite and Uno were the only islands where the trap catch was below 50% *An. melas* from indoor LTs. From outdoor LTs, only Uassa-Wite carried on this trend. A high level of hybridization between *An. gambiae s.s*. and *An. coluzzii* was seen on many islands, in particular from indoor LTs. Both *An. gambiae s.s*. and *An. coluzzii* were caught in small numbers throughout the islands.

**Table 2:** *Anopheles gambiae* species composition from indoor and outdoor LTs across the Bijagós Archipelago

Island	Indoor					Outdoor				
	*An. gambiae s.s.* (%)	*An. coluzzii (%)*	*An. gambiae*/*An. coluzzii* hybrids (%)	*An. melas (%)*	Total	*An. gambiae s.s.* (%)	*An. coluzzii (%)*	*An. gambiae*/*An. coluzzii* hybrids (%)	*An. melas* *(%)*	Total
Bubaque	2 (5.9)	0 (0.0)	7 (20.6)	25 (73.5)	34	2 (3.3)	2 (3.3)	11 (18.3)	45 (75.0)	60
Canhabaque	0 (0.0)	0 (0.0)	2 (7.4)	25 (92.6)	27	0 (0.0)	0 (0.0)	1 (1.6)	60 (98.4)	61
Canogo	1 (1.7)	3 (5.1)	2 (3.4)	53 (89.8)	59	2 (5.9)	1 (2.9)	2 (5.9)	29 (85.3)	34
Carache	0 (0.0)	0 (0.0)	0 (0.0)	4 (100.0)	4	0 (0.0)	0 (0.0)	0 (0.0)	1 (100.0)	1
Caravela	0 (0.0)	0 (0.0)	2 (18.2)	9 (81.8)	11	0 (0.0)	0 (0.0)	0 (0.0)	3 (100.0)	3
Formosa	6 (42.9)	1 (7.2)	4 (28.6)	3 (21.4)	14	1 (50.0)	0 (0.0)	0 (0.0)	1 (50.0)	2
Meneque	0 (0.0)	1 (4.2)	0 (0.0)	23 (95.8)	24	0 (0.0)	0 (0.0)	1 (3.3)	29 (96.7)	30
Nhago	0 (0.0)	0 (0.0)	0 (0.0)	19 (100.0)	19	2 (2.8)	0 (0.0)	0 (0.0)	70 (97.2)	72
Orango Grande	0 (0.0)	0 (0.0)	0 (0.0)	50 (100.0)	50	0 (0.0)	0 (0.0)	0 (0.0)	37 (100.0)	37
Rubane	2 (2.4)	0 (0.0)	3 (3.6)	79 (94.0)	84	6 (13.0)	1 (2.2)	0 (0.0)	39 (84.8)	46
Soga	3 (15.8)	0 (0.0)	2 (10.5)	14 (73.7)	19	0 (0.0)	0 (0.0)	3 (9.7)	28 (90.3)	31
Tchedega	3 (4.4)	0 (0.0)	3 (4.4)	62 (91.2)	68	0 (0.0)	0 (0.0)	0 (0.0)	5 (100.0)	5
Uassa-Wite	5 (31.2)	1 (6.2)	6 (37.5)	4 (25.0)	16	10 (43.5)	5 (21.7)	5 (21.7)	3 (13.0)	23
Unhocomo	0 (0.0)	0 (0.0)	0 (0.0)	0 (0.0)	0	0 (0.0)	0 (0.0)	0 (0.0)	2 (100.0)	2
Uno	5 (31.2)	1 (8.3)	4 (33.3)	2 (16.7)	12	0 (0.0)	0 (0.0)	0 (0.0)	12 (100.0)	12
Uracane	0 (0.0)	0 (0.0)	3 (50.0)	3 (50.0)	6	1 (8.3)	0 (0.0)	1 (8.3)	10 (83.3)	12

CSP-ELISA was conducted on a sample of 934 *An. gambiae* (434 from indoor LTs, 500 from outdoor LTs). Eight (SR 0.9%) specimens were found to be positive for CSP, seven of them were from outdoor LTs (SR 1.4%) with the remaining one caught indoors (SR 0.2%). All CSP-positive specimens were *An. melas* (Table 3). Seven positive *An. melas* were caught on Canogo, Nhago and Orango Grande from outdoor LTs; one was caught on Meneque from an indoor LT.

**Table 3:** Results from CSP-ELISA performed on *Anopheles gambiae* from across the Bijagós Archipelago

Larval collections	Total positive	Total negative	Infection rate (%)
*An. gambiae s.s*	0	55	0.0
*An. coluzzii*	0	15	0.0
*An. gambiae*/*An. coluzzii* hybrid	0	63	0.0
*An. melas*	8	761	1.0

In total 33 larval sites were sampled throughout the Bijagós, of which 13 (39.4%) contained *Anopheles* larvae (Fig. 1). In total, 2113 larvae were collected, of those, 727 were anopheline larvae and 1386 were culicine larvae. *Anopheles* larvae were most commonly found in natural larval sites with a perimeter of 1.01–10 m (Table 4). They were more regularly found in sites with direct sunlight and no vegetation. When vegetation was present, both grasses and trees (either fallen or standing) were common.

**Table 4:** Characteristics of *Anopheles*-positive larval sites

Variable	% of larval sites	% *Anopheles* larvae caught
Larval body type		
Natural	61.5	58.0
Artificial	38.5	37.6
Habitat perimeter		
0.01–1 m	38.5	17.4
1.01–10 m	61.5	68.2
10.01–100 m	0.0	0.0
> 100 m	0.0	0.0
Direct sunlight		
Yes	66.7	59.7
No	33.3	18.9
Vegetation present		
Yes	38.5	89.9
No	61.5	37.2
Vegetation type^a^		
Grasses, reeds or sedges	100.0	100.0
Trees	40.0	97.8

^a^Vegetation only found in five of the 12 larval sites

## Discussion

This survey provides, for the first time, descriptive data on the malaria vector population of 16 of the 18 permanently-inhabited islands of the Bijagós Archipelago. It reveals that vector species within *An. gambiae* are present throughout the islands. Thirteen of the 16 islands recorded a female *Anopheles* density of below ten per trapping night from both indoor and outdoor LTs. The islands with higher densities were all sampled in the latter stages of the survey, towards the end of November and beginning of December. Environmental variables have been documented to impact the density and species diversity of *An. gambiae* with rain being a major driver in mosquito numbers, providing water bodies for oviposition and larval development [18, 19]. There was a late-season rain event in mid-November, which may have resulted in a late surge in *Anopheles* on the islands of Nhago, Canhabaque and Orango Grande, all of which were sampled in late November or early December.

As well as environmental changes, ecological differences likely contributed to the variation in density between islands. The Bijagós is a UNESCO biosphere reserve, and has a variety of different landscapes, including palm groves, lakes, wetlands, rivers, savannahs, mangroves and gallery forests [20]. Areas with more sandy, well-drained soil may be unable to provide suitable bodies of water to sustain large immature vector populations [21]. Other landscapes have been shown to be closely associated with specific species, such as the presence of *An. melas* larvae in mangroves [22–25]. Studies using remote-sensed data to model the vector population on the Bijagós would be useful to explain some of the variations in island densities.

The island of Nhago was an outlier with densities of 98.7 per trapping night and 165.2 per trapping night from indoor and outdoor LTs, respectively, with PCR identifying the majority of the subsample analysed as *An. melas*. One of the largest and most productive larval bodies found during the survey was on Nhago, approximately 500 m outside of the village (larval site can be seen in Fig. 1). *Anopheles melas* are able to sustain a population throughout the dry season by ovipositing and developing as larvae in brackish water with a higher salinity than most other species within the *An. gambiae* complex [4].

*Anopheles melas* had previously been identified as being important in transmission in November/December on the island of Bubaque [8]. Relatively little contemporary information on the behaviour of *An. melas* is available, and different feeding behaviours have been described. In Liberia and Senegal, *An. melas* has been documented as being highly anthropophilic, whereas in Nigeria it is reported to be more opportunistic and less anthropophilic than other *An. gambiae* species [22, 24, 26]. The presence of *An. melas* within households from this survey indicates potential human host-seeking. All CSP-positive *An. gambiae* analysed were *An. melas*. The presence of *An. melas* outdoors and proportion of outdoor CSP-positive *An. melas* indicate that the species may contribute to residual transmission on the Archipelago, but future work is needed to better understand the *An. melas* population and its feeding-preferences. Malaria continues to be a problem across the islands, so understanding the roles of different vectors and how they might be targeted through interventions is important [27].

Species within *An. gambiae* have been described on mainland Guinea-Bissau, with studies identifying *An. gambiae s.s.* and *An. coluzzii* as the dominant malaria vectors [28–31]. A high level of hybridization between *An. gambiae s.s.* and *An. coluzzii* has also been described on mainland Guinea-Bissau [32, 33]. Vicente et al*.* showed that there was introgressive hybridization between the two species in coastal areas in mainland Guinea-Bissau [32]. Introgressive hybridization is when hybrid speciation arises through the accumulation of genetic material from parental lineages in an admixed population [34, 35]. This results in a population with high genetic diversity, enabling a distinct, ecologically divergent population to establish which may be able to adapt to new or marginal niches. In Guinea-Bissau, a stable population of hybrids has been established, with hybridization rates > 20% being recorded for almost 20 years [8, 32, 33, 36, 37]. Overall, hybrids accounted for 7.0% of *An. gambiae* caught throughout the survey (ranging from 0–50% from indoor LTs and 0–21.7% from outdoor LTs), accounting for more of the *Anopheles* caught than either of the parent species, following the same trend as seen in coastal regions of the mainland [32, 33, 37]. A survey looking at a panel of genetic markers in *Anopheles* mosquitoes caught on the islands and mainland to assess for evidence of genetic isolation between the two populations was conducted in 2012 [36]. Despite the distance between the mainland and the islands being greater than known *An. gambiae* dispersal capabilities (> 7 km with wind), it found no evidence of population isolation suggesting there is considerable gene flow [36, 38–40]. It is not surprising, therefore, that vector populations on the islands are comparable to those seen in similar ecosystems on the mainland. From the specimens sampled, no CSP-positive hybrids were identified, therefore, their contribution to malaria transmission from October to December is unknown.

Outdoor biting is a key driver of residual transmission [3]. ITN use is high in the Bijagós Archipelago, with 97% of participants surveyed indicating they slept under a net [41]. However, transmission persists regardless, indicating that the human population remains exposed to infective vectors. The survey results, which found similar densities of host-seeking female *Anopheles* outdoors and female *Anopheles* trapped indoors, support the hypothesis that outdoor biting plays a role in malaria transmission throughout the Archipelago. This is further reinforced by seven of the eight CSP-positive *Anopheles* mosquitoes being caught in outdoor LTs.

There are several limitations to the survey. Firstly, the use of different trapping techniques for indoor and outdoor LTs makes it difficult to draw conclusions about vector feeding behaviour. It is advised that the MB5 lure used for outdoor LTs is refrigerated prior to use [11]. Unfortunately, this was logistically infeasible in the Bijagós, therefore, lures were not refrigerated, which may have led to degradation in the lure quality over time. The terrain on the Bijagós made it difficult to have a systematic approach to larvae surveillance, and this was especially the case in thickly-forested areas. Collections were reliant on paths through the forest and local knowledge of potential larval sites. Some larval sites will have been missed, and it is, therefore, important to state this was not a comprehensive larval survey. More work needs to be done to better locate and characterize larval sites on the Bijagós. There was also not the capacity to rear larvae to adulthood and identify specimens to species level, therefore, it is unknown whether the larval sites located in the present study were associated with one species more frequently.

## Conclusions

Despite ITN use being high in the Bijagós Archipelago, malaria transmission persists, indicating that the human population remains vulnerable to infective bites. It is, therefore, important to thoroughly understand the vector population on the islands to better inform future control strategies. Here, details of vector density, species composition and infectivity rate from across the islands are presented for the first time. *Anopheles gambiae* s.l. was found throughout the Archipelago. Density and species composition varied between islands, with *An. melas* identified as playing a key role in transmission. This is highlighted by its occurrence at high percentages in both indoor and outdoor LTs on most of the islands and also the presence of the infective sporozoite stage in eight of the specimens analysed. Although the trapping technique was different between indoor and outdoor LTs, the outdoor density relative to that of indoor LTs suggests that outdoor biting is likely to play a role in residual transmission of malaria on the Archipelago.

## Data Availability

The dataset collected and analysed during the current study are available from the corresponding author on reasonable request.

## Abbreviations

ITN: Insecticide-treated nets
LT: CDC Light trap
RFLP: Restriction-fragment length polymorphism
CSP: Circumsporozoite protein
ELISA: Enzyme-linked immunosorbent assay
SR: Sporozoite rate
